# Circ_PUM1 promotes the development of endometrial cancer by targeting the miR‐136/NOTCH3 pathway

**DOI:** 10.1111/jcmm.15069

**Published:** 2020-02-19

**Authors:** Zhi‐Hong Zong, Yao Liu, Shuo Chen, Yang Zhao

**Affiliations:** ^1^ Department of Gynecologic Oncology Research Office The Third Affiliated Hospital of Guangzhou Medical University Guangzhou China; ^2^ Key Laboratory of Reproduction and Genetics of Guangdong Higher Education Institute in Guangdong Province Guangzhou China; ^3^ Department of Gynecology The First Affiliated Hospital of China Medical University Shenyang China

**Keywords:** circ_PUM1, circular RNA, endometrial cancer, microRNA, NOTCH3

## Abstract

Endometrial cancer is one of the most common gynaecological malignancies and the sixth most common cause of cancer‐related death among women. Here, we define the role and molecular mechanism of circ_0000043 (hereafter referred to as circ_PUM1) in the development and progression of endometrial carcinoma. QRT‐PCR was used to detect the expression of circ_PUM1 in normal endometrial tissue and endometrial carcinoma tissues. Changes in cell function and tumorigenicity in nude mice were examined after circ_PUM1 overexpression or knockdown. Bioinformatic analysis and dual‐luciferase reporter assay were used to predict and analyse the miRNAs that circ_PUM1 binds. Gene expression changes were analysed using Western blot. Circ_PUM1 was expressed at significantly higher levels in endometrial cancer tissues than in normal tissues. Up‐regulation of circ_PUM1 promoted the proliferation, migration and invasion of endometrial carcinoma cells. Opposite results were observed with circ_PUM1 knockdown, and the tumorigenic ability of endometrial cancer cells after circ_PUM1 knockdown was reduced compared to control cells. Circ_PUM1 is capable of binding to miR‐136, and up‐regulating its target gene NOTCH3, which can be reversed by overexpression of miR‐136. Circ_PUM1 can compete with miR‐136, leading to up‐regulation of NOTCH3, and thereby promote the development of endometrial cancer.

## 1 INTRODUCTION

Endometrial carcinoma is one of the most common gynaecological malignancies. According to the American Cancer Society, the number of new cases of endometrial cancer in the United States reached 61 380 in 2017, accounting for the fourth highest incidence (7%) of cancer in females, with an annual mortality close to 11 000. It is the sixth most common cause of cancer‐related death among women,^1^ with an increasing trend in recent years. The 5‐year survival rate of patients with stage I endometrial carcinoma is 96%, while that of stage IV patients is only 17%. Early diagnosis and treatment of endometrial cancer is critical for improving patient survival and quality of life.

Circular RNA, a covalently closed non‐coding RNA, is widely found in eukaryotic cell transcripts. Thousands of endogenous circular RNAs have been identified in mammalian cells, and the field has recently become a research hotspot. Circular RNAs are comprised of exons or introns generated by reverse shearing and other mechanisms, do not have 5' caps or poly(A) tail structures and are highly stable.^2^ These properties make circular RNAs a highly efficient and competitive endogenous RNA. Circular RNAs may function as sponges by adsorbing corresponding miRNAs,^3^ releasing expression of downstream target proteins and participating in various biological processes. It is reported that circRNAs play a pivotal role in the occurrence and development of a variety of cancers, including liver cancer, colon cancer, gastric cancer and others.^4^, ^5^, ^6^ We focus on a circRNA PUM1, which is reported to promote the malignant behaviour of lung adenocarcinoma and ovarian cancer,^7^, ^8^ but related studies in endometrial carcinoma have not yet been reported.

This research aimed to explore the role of circRNA PUM1 in the development and progression of endometrial cancer.

## 2 METHODS

### 2.1 Specimens

A total of 69 cases of endometrial carcinoma tissue specimens were collected from patients who underwent surgery from January 2015 to January 2017 in the First Affiliated Hospital of China Medical University (Shenyang, China); and 16 normal endometrial specimens were collected from the normal endometrial tissue of non‐menopausal patients with uterine fibroids undergoing hysterectomy. Two pathologists independently confirmed tumour specimens. No patients received preoperative chemotherapy or radiation therapy. The research was officially supported by the Ethics Committee of the China Medical University, and informed consent was obtained at the time of specimen collection. All specimens were processed and anonymized in accordance with ethical and legal standards.

### 2.2 Cell culture and transfection

Endometrial cancer cell line HEC‐1B and Ishikawa human endometrial carcinoma cells were separately cultured in high glucose DMEM medium and RPMI‐1640 medium containing 1% streptomycin and penicillin, and 10% foetal bovine serum (FBS). The cells were placed in a 5% CO_2_, 37°C incubator for maintenance culture, and the media were changed once every 24‐48 hours depending on cell confluence. When adherent cells approached confluence, they were digested with trypsin and subcultured. All operations of cell culture were performed under aseptic conditions in a clean hood, and all subsequent experiments were performed using cells in logarithmic growth phase. Cell transfection was performed using LipofectamineTM 3000 according to the manufacturer's instructions. The circ_PUM1 plasmid sequence is shown in Table S3.

### 2.3 Cell proliferation assay

Cell proliferation was analysed by MTT assay. The log phase cells were trypsinized and counted. 3 × 10^3^ cells were distributed in each well of 96‐well plates, along with 100 µL of culture media. The experiment was divided into two groups, the transfection negative control group and the transfection experimental group, and measurements were taken at 4 time‐points: 0, 24, 48 and 72 hours. At each time‐point, 20 µL MTT solution was added and cells were cultured at 37°C for 4 hours, the media were discarded, 150 µL DMSO was added, and the OD value was measured at 490 nm. The proliferation of cells in the negative control group and the experimental group was compared. Each experiment was repeated in triplicate.

### 2.4 Wound healing assay

A total of 10^6^ cells were added to each well of 6‐well plates with 2 mL of culture media, and allocated into a negative control group and experimental group. When cells were adherent, a 200 μL pipette tip was used to create a scratch. The cells were then incubated in a 37°C incubator with 5% CO_2_. The scratch widths were measured under an inverted microscope at 0, 24, and 48, and the scratch widths of the two group were compared at each time‐point. Cell mobility was quantified using the following equation: ([0 hour scratch width—scratch width after migration]/0 hour scratch width) ×100%. Experiments were performed in triplicate.

### 2.5 Invasion assay

The upper chamber surface of a transwell chamber was coated with 50 mg/L Matrigel at 1:10 dilution and placed in a 37°C incubator for polymerization for 4 hours. Cells were serum‐starved for 12‐24 hours, then trypsinized. 5 × 10^4^ ‐8 × 10^4^ cells were added to the upper chamber with 200 μL of serum‐free media. 600 μL of FBS‐containing culture media was added to the lower chamber to promote chemotaxis. The cells were divided into a negative control group and experimental group. Cells were incubated for 48 hours at 37°C in a 5% CO_2_ incubator. Media were then aspirated, and cells in the upper chamber were removed with a cotton swab. After fixing for 4 minutes in 4% paraformaldehyde and washing twice with PBS, the lower side of the chamber membrane was stained with 0.1% crystal violet for 20 minutes. The polycarbonate film from each chamber was then excised, attached to a glass slide, and covered with a cover glass. The number of cells was counted in several visual fields (5‐8), and the number of stained cells in the negative control group, and the experimental group was compared.

### 2.6 Apoptosis assay

A total of 10^4^ cells were added to each well of a 6‐well plate with 2 mL of culture medium. The cells were divided into a negative control group and a experimental group. After 48 hours, the cells were washed twice with PBS. Adherent cells were collected by trypsin digestion without EDTA, and then centrifuged at 376 *g* for 5 minutes. The cells were collected, centrifuged, washed with cold PBS, and centrifuged again at 376 *g* for 5 minutes. The supernatant was discarded, and 50 μL of 1× binding solution was added. 5 μL Annexin V–FITC and 5 μL propidium iodide (PE) was then added, mixed and incubated for 15 minutes at room temperature. 400 μL 1× binding buffer was then added, and apoptosis was measured by flow cytometry. Each assay was performed in triplicate.

### 2.7 qRT‐PCR

Total RNA was isolated from endometrial cancer cell lines using Trizol reagent; cDNA was reverse transcribed using myeloblastosis virus transcriptase and random primers (Takara) according to the manufacturer's instructions. Genes of interest were amplified by real‐time quantitative PCR using a SYBR Premix Ex Taq™ II Kit (Takara). The expression level of each target gene was normalized to that of 18s mRNA.

### 2.8 Western blotting

The total protein in a sample was collected with RNA lysate and subsequently quantified and denatured; proteins were separated by SDS‐PAGE and transferred to Hybond membrane (Amersham), followed by blocking for 1 hour with 3% bovine serum albumen. Then incubated with primary antibodies against NOTCH3 (1:1000; Proteintech, Proteintech Group) for 2 hours, washed with TBST buffer 3 times, incubated with HRP‐conjugated anti‐rabbit secondary antibodies for 2 hours, washed three times and visualized using ECL luminescence solution.

### 2.9 Dual‐luciferase reporter assay

Luciferase reporter assay was conducted to evaluate that miR‐136 has a binding site within the 3' untranslated region (UTR) of circ_PUM1. HEK293T cells were seeded into 24‐well plates and cotransfected with 50 nmol/L miR‐136, or negative control and 600 ng wild type circ_PUM1 3' UTR or mutant control dual‐luciferase vector. After 48 hours, the samples were measured for luciferase activity using a Dual‐Luciferase Reporter Assay System (Promega).

### 2.10 In vivo tumorigenesis model

4‐ to 5‐week‐old, 18‐20 g BALB/c female nude mice (n = 10) were purchased from Beijing Experimental Animal Center and reared in an SPF environment. 1 × 10^7^ HEC‐1B cells transfected with sh‐circ_PUM1(n = 5) or mock transfected (n = 5) and suspended in PBS were injected subcutaneously. The tumour volumes were measured every 3 days. After 4 weeks, the mice were killed, and subcutaneously implanted tumours and intraperitoneal metastatic lesions were excised, measured and photographed. All animal feeding and in vivo experimental procedures were carried out in accordance with the Regulations on Laboratory Animal Management of the Animal Experimental Department of China Medical University.

### 2.11 Statistical analysis

Data are presented as the mean ± SEM deviation from at least three separate experiments and were analysed using SPSS 20.0 software (SPSS Inc). Statistical comparison of means between groups was performed using Student's *t* test. All *P*‐values are two‐sided; *P* < .05 was considered statistically significant.

## 3 RESULTS

### 3.1 Circ_PUM1 is highly expressed in endometrial carcinoma tissues and endometrial cancer cell lines

**Figure 1:**
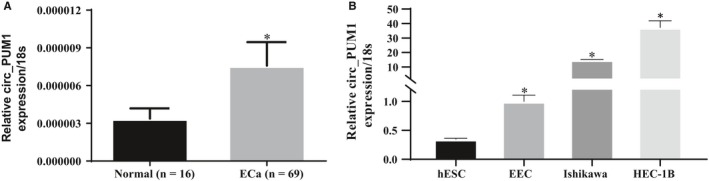
Circ_PUM1 is overexpressed in endometrial carcinoma. Circ_PUM1 was significantly higher in endometrial cancer than in normal endometrial tissue (A). The expression of Circ_PUM1 was highest in HEC‐1B, second in Ishikawa, third in EEC and lowest in hESC in endometrial cell lines (B). *P < .05

We detected the expression of circ_PUM1 by qRT‐PCR in 16 normal endometrial tissues and 69 endometrial carcinoma tissues. Circ_PUM1 expression was significantly higher in endometrial carcinoma than in normal endometrial tissue (Figure 1A, *P < .05), details could be found in Tables S1 and S2. We detected the expression of circ_PUM1 in normal endometrial stromal cell line (hESC), normal endometrial epithelial cell line (EEC) and endometrial cancer cell lines Ishikawa and HEC‐1B. The results showed that the expression of circ_PUM1 in EEC was higher than hESC, the expression of circ_PUM1 in Ishikawa was higher than EEC, and the expression of circ_PUM1 in HEC‐1B was higher than Ishikawa (Figure 1B, *P < .05).

### 3.2 Circ_PUM1 promotes proliferation and inhibits apoptosis of endometrial cancer cells

**Figure 2:**
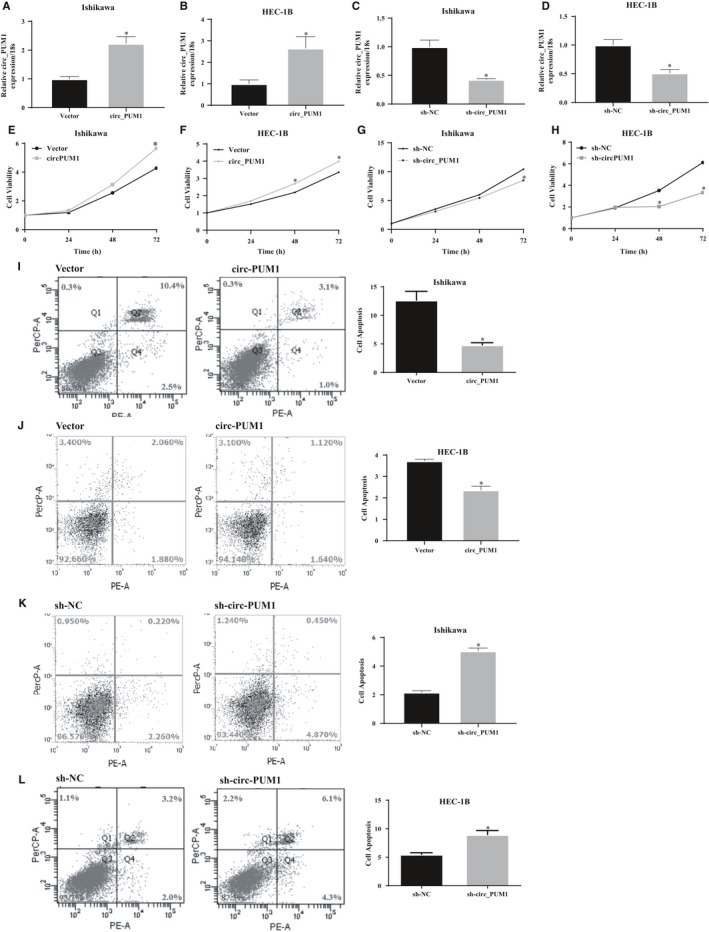
Circ_PUM1 promotes proliferation and inhibits apoptosis of endometrial cancer cells. After circ_PUM1 transfection, Ishikawa and HEC‐1B exhibited significantly higher circ_PUM1 expression (A,B); Silencing of circ_PUM1 resulted in lower circ_PUM1 expression (C,D). The up‐regulation of circ_PUM1 expression increased cell proliferation (E,F) and decreased apoptosis (I,J); Silencing of circ_PUM1 resulted in opposite effect (G,H and K,L). The results are representative of three separate experiments; *P < .05

Circ_PUM1 plasmid was transfected into endometrial cancer cells, and circ_PUM1 was knocked down in endometrial cancer cells by shRNA transfection (Figure 2A‐D, *P < .05). MTT and apoptosis assays showed that up‐regulation of circ_PUM1 expression increased cell proliferation (Figure 2E‐F, *P < .05) and decreased apoptosis (Figure 2I‐J, *P < .05). Furthermore, shRNA disrupted the circular structure of circ_PUM1, cell proliferation decreased (Figure 2G‐H, *P < .05) and apoptosis increased (Figure 2K‐L, *P < .05).

### 3.3 Circ_PUM1 promotes the migration and invasion of endometrial cancer cells

**Figure 3:**
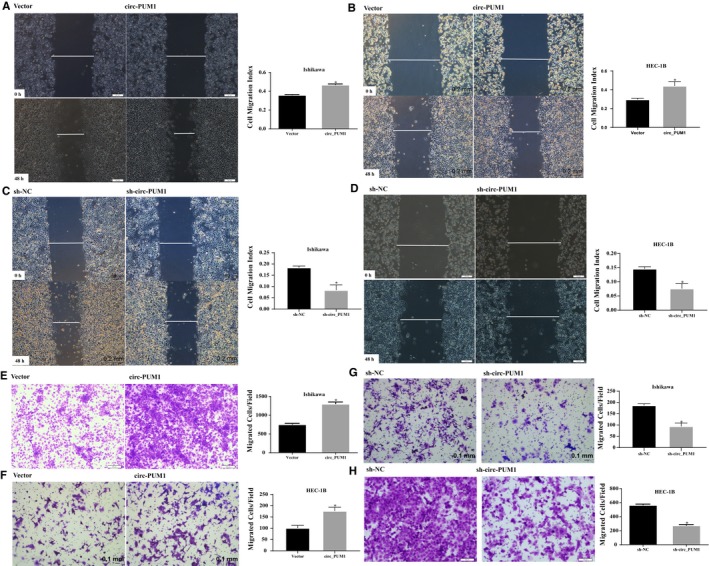
Circ_PUM1 promotes the migration and invasion of endometrial cancer carcinoma. Up‐regulation of circ_PUM1 expression increased cell migration (A,B) and invasion (E,F) in endometrial cancer cells. Silencing of circ_PUM1 resulted in opposite effect (C,D and G,H). The results are representative of three separate experiments; *P < .05

Through scratch assays and transwell migration experiments, we verified that cell migration and invasion were increased after up‐regulation of circ_PUM1 expression (Figure 3A,B,E,F, *P < .05). While, shRNA disrupted the circ_PUM1 loop structure, and cell migration and invasion capacity decreased (Figure 3C,D,G,H, *P < .05).

### 3.4 Disruption of circ_PUM1 structure decreases tumorigenic capacity of HEC‐1B cells in nude mice

**Figure 4:**
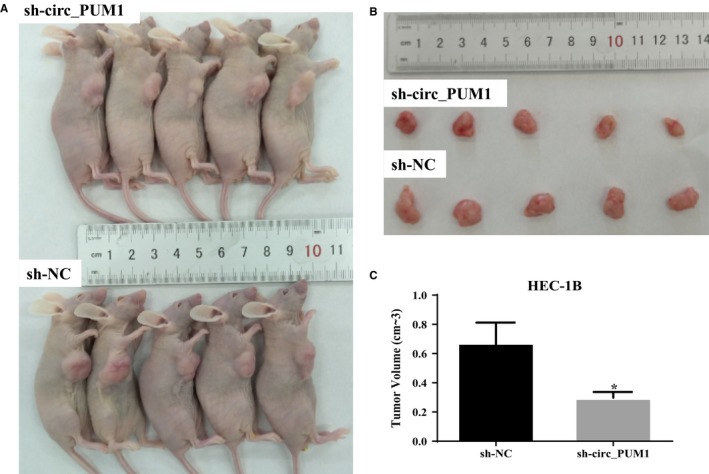
Effect of circ_PUM1 on in vivo tumour growth. Silencing of circ_PUM1 in HEC‐1B had a significantly smaller tumour formation volume than that in the control group which transfected with sh‐NC (A‐C). *P < .05

Compared with HEC‐1B cells transfected with sh‐NC, HEC‐1B cells transfected with sh‐circ_PUM1 showed significantly smaller tumour volumes and a relatively slow growth rate during the same time interval (Figure 4A‐C, *P < .05).

### 3.5 Circ_PUM1 is capable of functioning as a molecular “sponge” via binding to miR‐136

**Figure 5:**
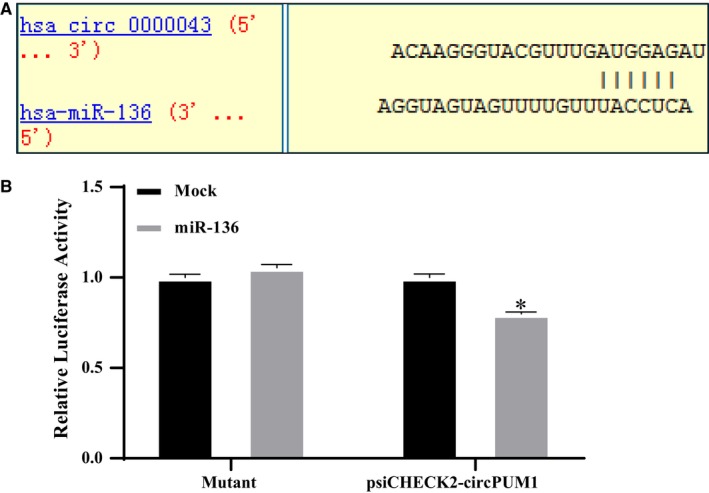
Circ_PUM1 directly binds to miR‐136. Bioinformatics software predicted the presence of a binding site for circ_PUM1 on miR‐136 (A). Circ_PUM1 bound directly to miR‐136 in HEK293 cells (B). The results are representative of three separate experiments; *P < .05

Bioinformatics software predicted miRNAs (Figure 5A) that may bind to circ_PUM1. Dual‐luciferase reporter gene results suggest that circ_PUM1 is capable of acting as a ‘sponge,’ binding to miR‐136 (Figure 5B, *P < .05).

### 3.6 Circ_PUM1 binds to miR‐136 and promotes downstream protein expression

**Figure 6:**
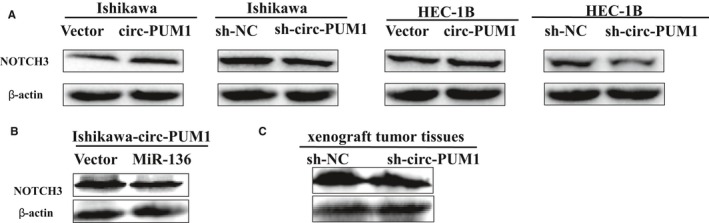
Circ_PUM1 promotes the development of endometrial cancer by regulating NOTCH3 proteins via sponges miR‐136.Overexpression of circ_PUM1 increased the expression of NOTCH3, and vice versa (A). High expression of miR‐136 in Ishikawa cells overexpressing circ_PUM1 resulted in a decrease in NOTCH3 expression (B); In nude mouse tumour tissue, low expression of irc_PUM1 inhibited levels of NOTCH3 (C). The results are representative of three separate experiments

Because circ_PUM1 binds directly to miR‐136, and miR‐136 binds directly to NOTCH3,^9^ we hypothesized that circ_PUM1 could promote tumorigenesis by promoting NOTCH3 expression via acting as a sponge for miR‐136. Western blot analysis showed that overexpression of circ_PUM1 promoted the expression of NOTCH3, while circ_PUM1 knockdown suppressed the expression of NOTCH3 (Figure 6A). Up‐regulation of miR‐136 in Ishikawa cells overexpressing circ_PUM1 resulted in a decrease in NOTCH3 expression (Figure 6B). Western blot analysis of mouse tumour tissue from the nude mouse model of tumorigenesis revealed that overexpression of circ_PUM1 increased NOTCH3 levels in vivo (Figure 6C). Together, these data suggest that circ_PUM1 promotes the development of endometrial carcinoma by targeting protein NOTCH3 via miR‐136.

## 4 DISCUSSION

Due to the lack of a free 3' or 5' end, circRNA is resistant to the normal mechanisms of linear RNA decay and therefore has a long half‐life.^10^ Due to the development and extensive use of RNA sequencing technology, especially those that do not rely on poly(A) purification,^11^ the expression profiles and biological functions of circRNAs have become a hot topic. Studies have shown that dysregulation of circRNAs is seen in a variety of cancers,^12^, ^13^, ^14^ but studies in gynaecological malignancies are rare.

We screened for abnormally expressed circRNAs in normal ovarian and ovarian carcinoma tissues by microchips. Among the identified circRNAs, circ_PUM1 was significantly up‐regulated in ovarian carcinoma tissues.^8^ We wondered whether the circRNA was also expressed in endometrial cancer. The expression of circ_PUM1 was detected by qRT‐PCR in 16 normal endometrium and 69 endometrial cancer tissues. These results confirmed that circ_PUM1 is expressed in endometrial cancer and normal endometrium, and that expression in cancer is significantly up‐regulated relative to normal endometrial tissue. Meanwhile, the expression of circ_PUM1 in endometrial cancer cells was significantly higher than that in normal endometrial epithelial cells and endometrial stromal cells.

To further investigate the role of circ_PUM1 in endometrial cancer, we examined its expression in HEC‐1B, and Ishikawa cells, and found that it was most highly expressed in HEC‐1B and least abundant in Ishikawa cells. Furthermore, overexpression plasmids and shRNAs targeting circRNA cleavage sites were designed. The expression plasmid and shRNA were stably transfected in Ishikawa and HEC‐1B to evaluate effects on cell proliferation, apoptosis, migration and invasion capacity. We found that cells overexpressing circ_PUM1 showed a significantly accelerated proliferation rate, decreased apoptosis rate, and increased migration and invasion capacity compared with the control group. Opposite findings were observed with circ_PUM1 knockdown in Ishikawa and HEC‐1B cells. HEC‐1B cells with circ_PUM1 knockdown were injected subcutaneously into nude mice, and the tumour volume was significantly smaller than that of the control group at the same time‐point. Together, these data suggest that circ_PUM1 plays a role in the development of endometrial cancer.

Studies have shown that circRNAs play an important role in gene regulation at the transcriptional or post‐transcriptional level,^15^ and their role in regulating non‐coding microRNAs (miRNAs) has become a focus of intensive research in recent years. MiRNAs are a class of small non‐coding RNAs that match the sequence of the 3'‐UTR seed of a target gene mRNA. Complementary pairing inhibits translation of the target mRNA or induces its degradation.^16^ CircRNAs are highly stable and rich in miRNA reaction elements (MREs), making them highly efficient as miRNA ‘sponges’.^3^, ^16^, ^17^ For example, ciRS‐7 contains more than 69 selectively conserved miRNA target sites that strongly inhibit miR‐7 activity.^18^ We wondered if circ_PUM1 also carries this function and used a bioinformatics approach to predict its miRNA binding sites. We analysed the predicted miRNAs and their corresponding downstream proteins in detail, combined with dual‐luciferase reporter assay for verification. Our results show that miR‐136 can bind to circ_PUM1.

The role of miRNAs in gene dysregulation in cancer is critical.^19^, ^20^, ^21^ MiR‐136 has also been studied in various cancers. MiR‐136 has been identified as a tumour suppressor gene in various adenocarcinomas such as breast cancer, colon cancer and lung cancer.^22^, ^23^, ^24^ In addition, miR‐136 can inhibit the activity of ovarian cancer stem cells and enhance the sensitivity of ovarian cancer to paclitaxel by targeting NOTCH3.^9^

Notch signalling affects a variety of cellular processes, including maintenance of undifferentiated states, participating in cell fate decisions, inducing terminal differentiation and other functions involved in the progression of cancer.^25^ Notch was originally found to be an oncogene in T cell leukaemia 26 and mediates TGF‐α‐induced changes in epithelial differentiation during pancreatic tumorigenesis.^27^ NOTCH3 is more strongly expressed in endometrial cancer cells than in normal endometrial gland cells and is associated with poor prognosis.^28^

Based on these data, we examined changes in NOTCH3 expression in Ishikawa and HEC‐1B after overexpressing circ_PUM1 or knocking out circ_PUM1 by Western blot. We found that up‐regulation of circ_PUM1 promoted NOTCH3 protein expression compared with the control group, while circ_PUM1 knockdown yielded the opposite result. Up‐regulation of miR‐136 in Ishikawa cells overexpressing circ_PUM1 reversed the up‐regulation of NOTCH3 protein by circ_PUM1.

In conclusion, our data suggest that circ_PUM1 plays a critical role in the development and progression of endometrial cancer, mainly by adsorbing miR‐136 via a ‘sponge’ effect and thereby promoting expression of the target gene NOTCH3. To the best of our knowledge, circ_PUM1 is the first circRNA to be studied in endometrial cancer and shown to be differentially expressed and functionally important. This research will provide new directions for improving and expanding molecular targets for the treatment of endometrial cancer.

## CONFLICTS OF INTEREST

The authors have no conflicts of interest to declare.

## AUTHORS' CONTRIBUTIONS

Yang Zhao conceived the study and analysed interpretation. Zhi‐Hong Zong performed the experiments, analysed data and wrote the manuscript. Yao Liu and Shuo Chen performed the experiments and analysed the data. All authors read and approved the final manuscript.

## DECLARATIONS

Ethics approval and consent to participate. The research protocol was approved by the China Medical University Ethics Committee (No: 2016‐32‐2).

## Data Availability

The datasets used and/or analysed during the current study available from the corresponding author on reasonable request.
