# Supplementary material for: Circ_PUM1 promotes the development of endometrial cancer by targeting the miR‐136/NOTCH3 pathway
Source: J Cell Mol Med. 2020 Feb 19;24(7):4127–35. doi: 10.1111/jcmm.15069 (PMC7171399; doi:10.1111/jcmm.15069)
Supplement: Supplementary file 1 [file JCMM-24-4127-s001.doc]

**Supplementary Table 1:** Circ-PUM1 expression in Normal endometrium and Endometrial cancer tissues

| **Groups** | **N** | **Circ-PUM1 expression / 18s** | ***P* value** |
| --- | --- | --- | --- |
|
| Normal endometrium | 16 | 3.376E-06 ± 8.117E-07 |  |
| Endometrial cancer | 69 | 7.586E-06 ±1.866E-06 | ***0.021*** |

Bold and Italics means P < 0.05.

**Supplementary Table 2:** Correlation of Circ-PUM1 expression with different clinicopathological features of Endometrial cancer

| **Clinicopathological features** | **N** | **Circ-PUM1 expression / 18s** | ***P* value** |
| --- | --- | --- | --- |
|
| **The pathology types** |  |  | ***0.003*** |
| Endometrioid adenocarcinoma | 54 | 4.764E-06±7.376E-07 |  |
| The other pathology types | 15 | 1.774E-05±7.81E-06 |  |
| **Age** |  |  | 0.228 |
| ≤ 55 | 30 | 5.004E-06±9.174E-07 |  |
| > 55 | 39 | 9.571E-06±3.209E-06 |  |
| **FIGO stages** |  |  | *>0.05* |
| I | 45 | 7.695E-06±2.520E-06 |  |
| II | 9 | 1.500E-06±5.819E-07 |  |
| III | 13 | 1.146E-05±4.509E-06 |  |
| IV | 2 | 7.304E-06±4.560E-06 |  |
| **Pathology classification** |  |  | *>0.05* |
| G1 | 34 | 5.077E-06 ±1.039E-06 |  |
| G2 | 11 | 3.850E-06 ±1.078E-06 |  |
| G3 | 9 | 4.696E-06 ±1.687E-06 |  |
| Bold and Italics means P < 0.05. The pathology classification is based on tissue specimens whose pathological type is endometrioid adenocarcinoma. | | | |

**Supplementary Table 3:** The RNA sequence and primer sequences of circPUM1.

>hsa_circ_0000043|NM_014676|PUM1|438bp

GGCCCAAGGGATGCAGACAGTGATGAAAACGACAAAGGTGAAAAGAAGAACAAGGGTACGTTTGATGGAGATAAGCTAGGAGATTTGAAGGAGGAGGGTGATGTGATGGACAAGACCAATGGTTTACCAGTGCAGAATGGGATTGATGCAGACGTCAAAGATTTTAGCCGTACCCCTGGTAATTGCCAGAACTCTGCTAATGAAGTGGATCTTCTGGGTCCAAACCAGAATGGTTCTGAGGGCTTAGCCCAGCTGACCAGCACCAATGGTGCCAAGCCTGTGGAGGATTTCTCCAACATGGAGTCCCAGAGTGTCCCCTTGGACCCCATGGAACATGTGGGCATGGAGCCTCTTCAGTTTGATTATTCAGGCACGCAGGTACCTGTGGACTCAGCAGCAGCAACTGTGGGACTTTTTGACTACAATTCTCAACAACAG

hsa_circ_0000043（Divergent primers）

hsa_circ_0000043-F: 5'- gcatggagcctcttcagtttg -3'

hsa_circ_0000043-R: 5'- ccattggtcttgtccatcac -3'

The RNA-sequencing data analysis of circ_PUM1 PCR electrophoresis product:


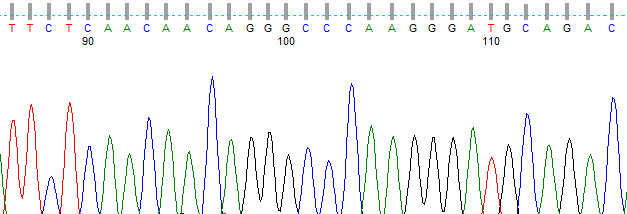
ring-forming splicing sites
